# Clinical Evaluation of Autologous and Allogeneic Stem Cell Therapy for Intrauterine Adhesions: A Systematic Review and Meta-Analysis

**DOI:** 10.3389/fimmu.2022.899666

**Published:** 2022-07-04

**Authors:** Jia-ming Chen, Qiao-yi Huang, Wei-hong Chen, Shu Lin, Qi-yang Shi

**Affiliations:** ^1^ Department of Gynecology and Obstetrics, The Second Affiliated Hospital of Fujian Medical University, Quanzhou, China; ^2^ Center of Neurological and Metabolic Research, The Second Affiliated Hospital of Fujian Medical University, Quanzhou, China; ^3^ Group of Neuroendocrinology, Garvan Institute of Medical Research, Sydney, NSW, Australia

**Keywords:** meta-analysis, intrauterine adhesion, autologous stem cells, allogeneic stem cells, immunological rejection

## Abstract

**Objective:**

Intrauterine adhesions (IUAs) are a major cause of female infertility. Stem cells can be used to restore endometrial function owing to their regenerative abilities. We compared the safety and efficacy of autologous and allogeneic stem cell treatments in patients with recurrent IUA after conventional therapy based on a systematic review of the related literature.

**Methods:**

The PubMed, Embase, and Cochrane databases were systematically searched. All analysis were performed using Review Manager 5.4. We compared improvements in endometrial thickness, pregnancy rates, menstruation, and side effects after autologous and allogeneic stem cell therapy. The study was registered with PROSPERO, CRD 42022322870.

**Results:**

Our search returned 154 reports, 10 of which met the inclusion criteria, representing 116 patients. Of these, 44 patients in two studies were treated with allogeneic stem cells and 72 patients in eight studies were treated with autologous stem cells. Improvements in endometrial thickness and pregnancy rates after intrauterine device treatment were compared between the autologous and allogeneic stem cell groups. Endometrial thickness increased more after autologous stem cell IUA treatment (mean difference, 1.68; 95% confidence interval [CI]: 1.30–2.07; P < 0.00001), and the pregnancy rate was also improved (relative risk, 1.55; 95% CI: 1.19–2.02, P < 0. 001). No obvious and serious adverse reactions were observed during stem cell therapy in either group.

**Conclusions:**

This meta-analysis and systematic review of the results of randomized trials of autologous and allogeneic stem cell treatments for IUA suggests that autologous stem cells have a better effect in improving the endometrium thickness and pregnancy rate.

**Systematic Review Registration:**

https://www.crd.york.ac.uk/PROSPERO/, identifier CRD42022322870.

## 1 Introduction

Intrauterine adhesions (IUAs) are a type of endometrial fibrosis caused by the invasion of exogenous pathogens after endometrial injury. Once the endometrial basal layer is damaged, fibrous tissue forms and the interstitial tissue disappears. Further, tissue bridges connect across all directions of the uterine wall, forming adhesions (1). Patients with IUA often have oligomenorrhea, amenorrhea, a thin endometrium, few glands, and can even suffer from infertility (2). Dilation and curettage after miscarriage is the major pathogenic factor of IUA, with an incidence ranging from 15% to 40% (3). In addition, an increasing number of cases IUA have been associated with abdominal and hysteroscopic myomectomy, diaphragmatic resection, and other types of intrauterine surgery (4). Asgari et al. (5) found that the incidence of postoperative adhesion in the laparoscopic group and open-surgery group was 21% and 19%, respectively. Moreover, Laganà et al. (6) performed a prospective study including 38 and 24 patients who underwent laparoscopic and caesarean myomectomy, respectively; 19.4% of the women in both groups developed IUA. Severe endometrial dysfunction can lead to this reproductive defect in women of childbearing age, which was once considered to be a terminal condition causing infertility (7).

The main purpose of clinical treatment for IUA is to restore the endometrial morphology, improve endometrial function, and prevent re-adhesion (8). Hysteroscopic endarterectomy is the most common treatment for IUA. One study found that the re-adhesion rate was 30% in cases of mild and moderate IUA and was 62.5% in cases of severe IUA. In addition, the ratio of pregnancy following treatment was only 22.5–33.3%, which is not satisfactory (9).

In recent years, stem cells have been found to have self-renewal and multi-directional differentiation potential, with broad prospects for the treatment of tissue damage involving the uterine cavity (10). Allogeneic stem cells are widely used in experiments because of their convenient extraction and easy access. However, owing to ethical considerations and immune rejection issues, autologous stem cells remain the first choice for human clinical applications.

Mesenchymal stem cells (MSCs) act as conducting cells, regulating host cells *via* signals delivered through extracellular vesicles (Evs) or exosomes (Exo), thereby improving liver fibrosis and promoting regeneration (11). Through animal experiments, Yao et al. (12) found that Exos from bone marrow-derived MSCs (BMSCs) might regulate the repair of the damaged endometrium through the transforming growth factor-β1/SMAD signaling pathway. Therefore, the concept of cell-derived Evs or Exo-based acellular therapy has been attracting attention. Cell-free therapy can be safely administered in high doses, resulting in the infiltration of target organs. However, Exos are rarely used in clinical practice at present. Cao et al. (13) and Zhang et al. (14) used allogeneic umbilical cord blood MSCs to treat IUA, and found that patients exhibited increased menstrual flow, increased endometrial thickness, and increased pregnancy rates. Moreover, Lee et al. (15), Santamaria et al. (16), and Singh et al. (17) found that autologous stem cell therapy could also improve these indicators.

There are currently no systematic reviews and meta-analysis evaluating the efficacy and safety of reported clinical trials of autologous and allogeneic stem cells for the treatment of patients with IUA. Therefore, we performed a meta-analysis to evaluate these clinical outcomes, including menstrual improvement, pregnancy, and changes in endometrial thickness, after the treatment of autologous or allogeneic stem cells, as well as the side effects of each type of treatment. In the analysis, each patient was used as their own control before and after treatment to compare the autologous stem cell group and allogeneic stem cell group.

## 2 Materials and Methods

### 2.1 Search Strategies

We registered our study on PROSPERO (CRD 42022322870). System Review and Meta-Analysis is the preferred reporting item based on the System Review and Meta-Analysis (PRISMA) guidelines (18). Two authors (JMC and QYH) independently systematically searched the PubMed, Embase, and Cochrane databases (until January 21, 2022) for relevant studies. We jointly determined the Medical Subject Heading (MeSH) terms and other search terms for article retrieval. If there was a dispute, the two authors negotiated until reaching a consensus or invited a third author (SL) to make a final judgment. The final search strategy was as follows: stem cell [MeSH Terms] and uterus synechia [MeSH Terms]. The search results of other free text and MeSH terms are provided in the **Supplementary Material**.

### 2.2 Study Selection Criteria

#### 2.2.1 Inclusion Criteria

The inclusion criteria were as follows: (a) patients with primary or secondary infertility, hypomenorrhea, or amenorrhea and IUA who had experienced one or more hysteroscopic operations; (b) all patients with IUA treated with autologous or allogeneic stem cells; (c) complete results, including at least one outcome of IUA score, endometrial thickness, menstrual volume, pregnancy rate, and other indicators; (d) all results consistent with the design and reporting of prospective and randomized controlled studies.

#### 2.2.2 Exclusion Criteria

Exclusion criteria were as follows: (a) duplicate studies in the three databases; (b) the selected subjects were animals such as mice, rats, and rabbits, among others; (c) the selected object was a review, or the study itself did not include a control group; (d) despite matching with the MeSH terms and free words, the content was not specific to stem cell therapy for IUA; (e) the title and abstract were relevant to the topic, but the full text could not be found.

### 2.3 Data Extraction

Research selection and data extraction were conducted by two independent reviewers (JMC and QYH). Any objection was discussed and submitted to a third reviewer (SL) for confirmation. The extracted content from each article included the (a) first author, year, and country of the study; (b) age, main symptoms, cause, and previous treatment of the patients; (c) changes in IUA score, endometrial thickness, pregnancy rate, and menstrual volume before and after treatment; (d) stem cell type (autologous stem cells or allogeneic stem cells) and stem cell therapy dose; (e) postoperative white blood cell count; and (f) side effects such as headache, nausea, and vomiting, among others.

### 2.4 Quality Assessment

The ROBINS tool was used to assess the methodological quality and risk of bias of the included trials according to the following eight areas: confounding factors (all outcomes), selection, classification, deviations from intended interventions (assignment), missing data (all outcomes), measurement of outcome (all outcomes), selection of reported result (all outcomes), and overall (19).

### 2.5 Statistical Analysis

All meta-analysis were performed using Review Manager 5.4 (Cochrane Collaboration, London, UK). First, according to the comparison before and after stem cell treatment, the patients were divided into a treatment and control group. The main binary variables were menstrual improvement after treatment and pregnancy, among others, represented by the relative risk (RR) and 95% confidence interval (CI). Continuous variables were as follows: change in endometrial thickness, represented by the mean difference. Second, according to the type of stem cells, patients were divided into autologous and allogeneic stem cell groups.

The Q-test obeyed the chi-square (χ^2^) distribution of degrees of freedom. The chi-square value and inconsistency index (I^2^) were used to evaluate the heterogeneity of each study. An I^2^ value of 50% indicates moderate heterogeneity and an I^2^ > 75% is highly heterogeneous. When I^2^ < 50%, the fixed-effects model was adopted. When I^2^ > 50%, subgroup analysis and sensitivity analysis were performed using a random-effects model.

## 3 Results

### 3.1 Search Results

**Figure 1:**
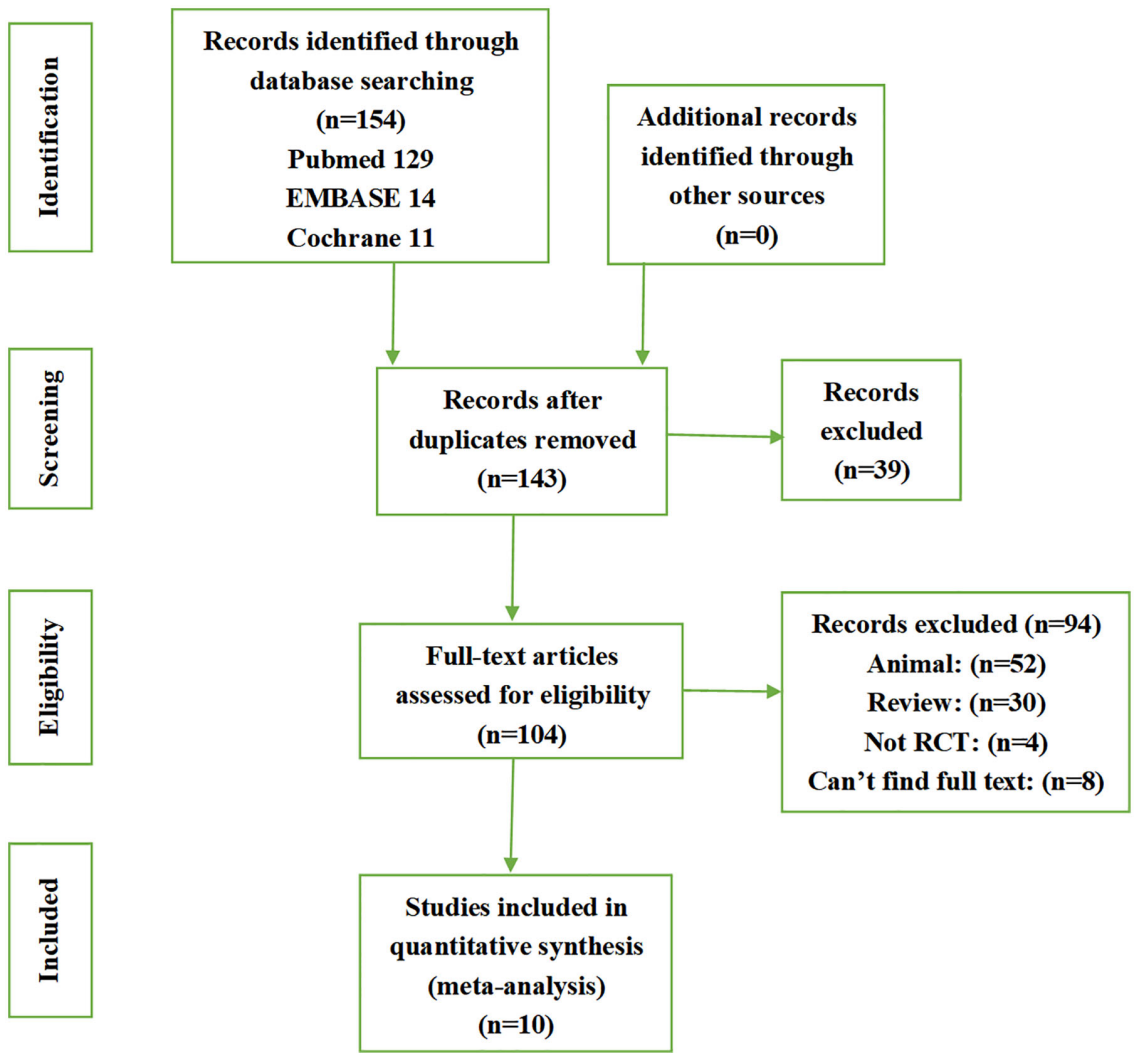
Flow chart of search results.

In total, 154 articles were retrieved, including 129 from PubMed, 14 from Embase, and 11 from the Cochrane database. All of the retrieved articles were imported into Endnote, with 11 duplicates found. A closer look at the titles and abstracts revealed 30 reviews and 52 animal experiments. Full-text review identified four articles that were not randomized controlled trials, and the full text could not be found for eight articles, likely because the clinical study had not been completed. Finally, a total of 10 studies were included in the analysis, including two studies using allogeneic stem cells to treat IUA (13, 14) and eight studies using autologous stem cells to treat IUA (15–17, 20–24). The flow chart of the search process is shown in **Figure 1**.

### 3.2 Characteristics of the Included Studies

The 10 studies included for final analysis reported on 116 patients in total, 72 of whom received autologous therapy and 44 of whom who received allogeneic therapy. The basic information of these patients is presented in **Tables 1**, **2**. These studies all took place from 2011 to 2021, with patients ranging in age from 20 to 45 years. The main symptoms were infertility and decreased menstrual volume. These patients had undergone one or more curettages and had received IUD lysis and estrogen therapy. However, these treatments were all ineffective. Stem cells combined with hormone therapy were used in all cases, and the menstrual recovery, endometrial repair, and pregnancy rate after treatment were recorded after a follow-up of 3 months to 5 years. In patients receiving allogeneic stem cell therapy, the postoperative white blood cell count was also recorded; however, this was not mentioned in the studies of patients treated with autologous stem cells.

**Table 1:** Characteristic of basic information.

Author	Country	Year	Age	Stem Cells	Patients	Symptoms	Etiology	Prior repair attempts	Reference
Cao et al	China	2018	27-42 (35.1±3.8)	allogeneic UC-MSC	26	Infertility Hypomenorrhea	D&C spontaneous abortion	HSP	(13)
Lee et al	Korea	2020	36-43 (39.2±2.8)	autologous ADSCs	5	Infertility	D&C Unexplained	HSC adhesiolysis HT	(15)
Santamaria et al	Spain	2016	30-45 (38.0±4.8)	autologous BMDSCs	11	Scant spotting Amenorrhea	D&C Unexplained IU	HSP	(16)
Sing et al	India	2014	25-35 (29.8±3.4)	autologous MNC	6	Infertility Amenorrhea	D&C	Hysteroscopic adhesiolysis HT	(17)
Zhang et al	China	2021	30-39 (34.1±3.6)	allogeneic UC-MSC	18	Infertility Hypomenorrhea	D&C HSP	HSA	(14)
Zhao et al	China	2017	20-38 (31.0±6.6)	autologous BMNCs	5	Infertility Hypomenorrhea	D&C	HSP	(24)
Nagori et al	India	2011	33	autologous BMDSCs	1	Infertility Hypomenorrhea	D&C	HSP	(21)
Tan et al	China	2016	20-40 (33.7±1.5)	autologous menSCs	7	Infertility	Spontaneous abortion, Artificial abortion, Intestinal tuberculosis, lymphatic tuberculosis, Hysteroscopic surgery	Adhesiolysis IUD HRT	(23)
Singh et al	India	2020	24-38 (29.6±4.1)	autologous BMNCs	25	Amenorrhea Scanty	TB D&C	Hysteroscopic adhesiolysis HT	(22)
Ma et al	China	2020	22-40 (35.8±3.6	autologous menSCs	12	Refractory IUA infertility	Curettage Unexplained Infection	Adhesiolysis	(20)

**Table 2:** Characteristics of treatment.

Author	Year	Stem Cells	Cell number	Combination Therapy	Follow-up	Postoperative leukocyte	Reference
Cao et al	2018	allogeneic UC-MSC	1×10^7 (4.2×10^5/cm2)	Progynova Progesteron	30 months	WBC:5.96 ± 1.46×10^9/L neutrophil percentage: 52.2 ± 8.99% C-reactive protein:2.27 ± 0.43 mg/L	(13)
Lee et al	2020	autologous ADSCs	4.6±0.7 ×10^6	Estradiol valerate Medroxyprogesteron	23 months	no mention	(15)
Santamaria et al	2016	autologous BMDSCs	123.56 × 10^6 (42-200×10^6 )	HRT	6 months	no mention	(16)
Sing et al	2014	autologous MNC	103.3×10^6±20.45	Taxim-O (cefexime, alkem pharma) Estradiol valerate Medroxyprogesterone	3, 6, 9 months	no mention	(17)
Zhang et al	2021	Allogeneic UC-MSC	1 × 10^7/mL (2 mL)	Estradiol valera Progesterone Dydrogesterone tablets Progesterone Soft Capsules	27 months	leukocyte: 6.69 ± 1.22×10^9/L lymphocyte: 2.37 ± 0.46×10^9/L neutrophils: 54.76 ± 6.74%	(14)
Zhao et al	2017	autologous BMNCs	1 ×10^6	Progynova	3 months	no mention	(24)
Nagori et al	2011	autologous BMDSCs	0.8mL	Progesterone vaginal gel Ethinyloestradiol Aspirin	6 months	no mention	(21)
Tan et al	2016	autologous menSCs	1×10^6	Oestradiol valerate Progesterone	3, 4, 6 months	no mention	(23)
Singh et al	2020	autologous BMNCs	65.3×10^6±37.2 19-200×10^6	Estradiol valerate Medroxyprogesterone	3, 6, 9 months, 5 years	no mention	(22)
Ma et al	2020	autologous menSCs	10×10^6	Estradiol Dydrogesterone	No mention	no mention	(20)

### 3.3 Methodologic Quality and Risk of Bias

**Figure 2:**
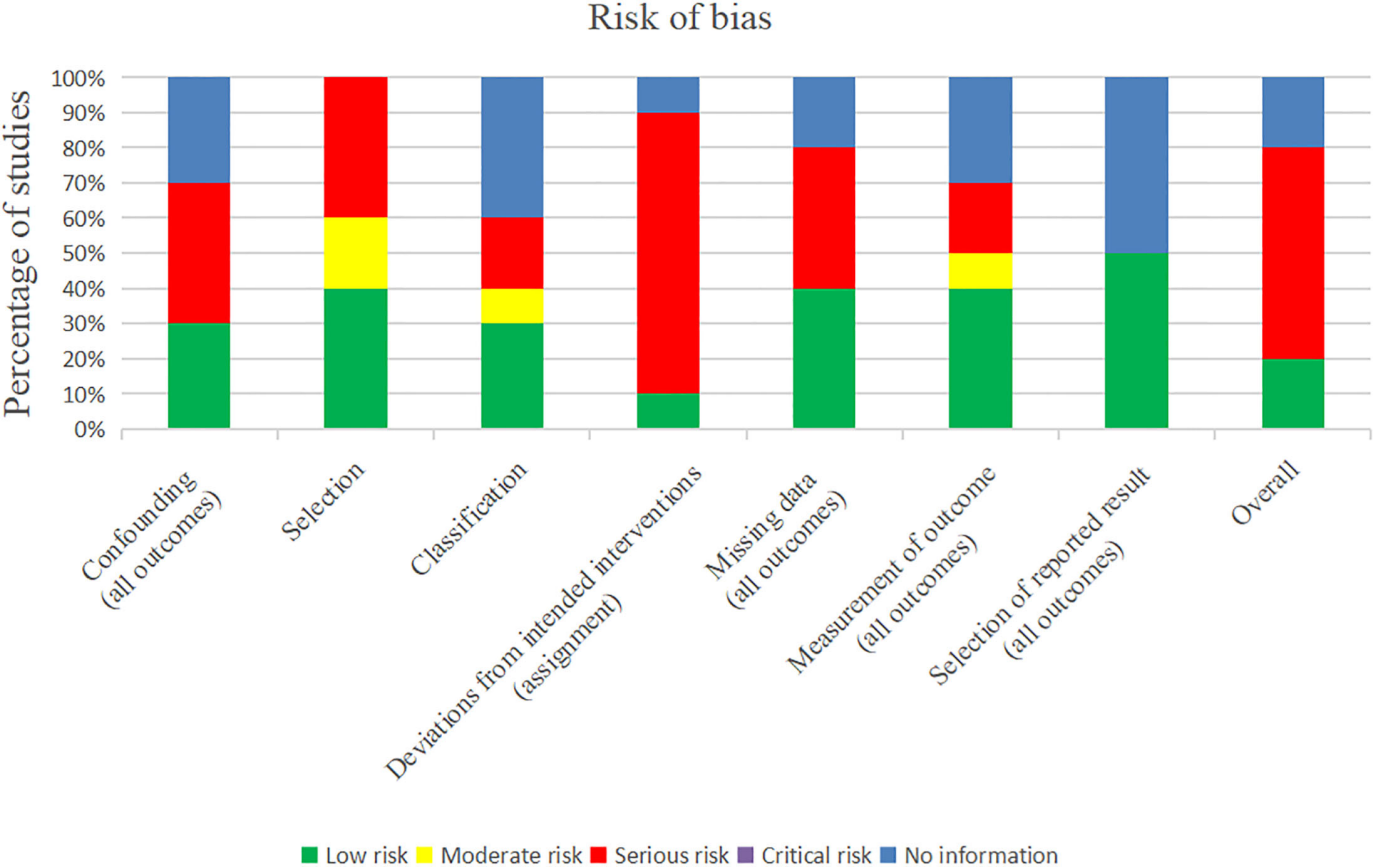
Risk of bias.

The ROBINS tool was used to evaluate the overall performance of the included studies from eight aspects (**Figure 2**). By analyzing each study, we found a serious risk of bias for four studies based on confounding factors, four studies based on selection, two studies based on classification, eight studies based deviations from intended interventions, and one study based on measurement of outcome. A low risk of bias was found for three studies based on confounding factors, four studies based on selection, three studies based on classification, one study based deviations from intended interventions, four studies based on missing data, four studies based on measurement of outcome, five studies based on selection of reported result, and two studies based on overall bias. The other studies had no related information or were judged to be at a moderate risk of bias. In addition, we generated a funnel plot in Review Manager 5.4 to analyze the possibility of publication bias and heterogeneity in the improvement of the endometrium after autologous stem cell treatment and the comparison of pregnancy rates. The results showed that the funnel plot was symmetrical, indicating low sensitivity to publication bias.

### 3.4 Meta-Analysis

#### 3.4.1 Overall Efficacy of Autologous and Allogeneic Stem Cell Therapy

##### 3.4.1.1 Menstrual Improvement

**Figure 3:**
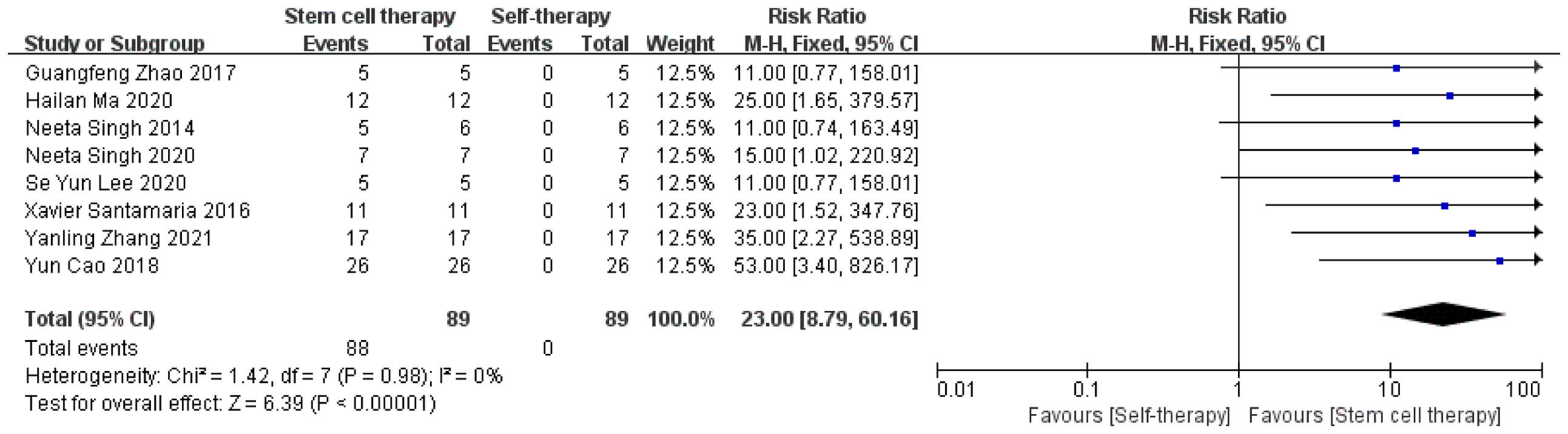
Menstruation improvement.

Of the 10 included studies, menstrual improvement was mentioned in eight studies with a total of 89 patients. Menstrual improvement was evaluated by comparing the changes in the patients’ own menstrual volume before and after the application of stem cell therapy for IUA. The results showed statistical significance (**Figure 3**). In addition, there was no statistically significant heterogeneity among the included trials (χ² = 1.42, df = 7, *P =* 0.98; I² = 0%).

##### 3.4.1.2 Pregnancy

**Figure 4:**
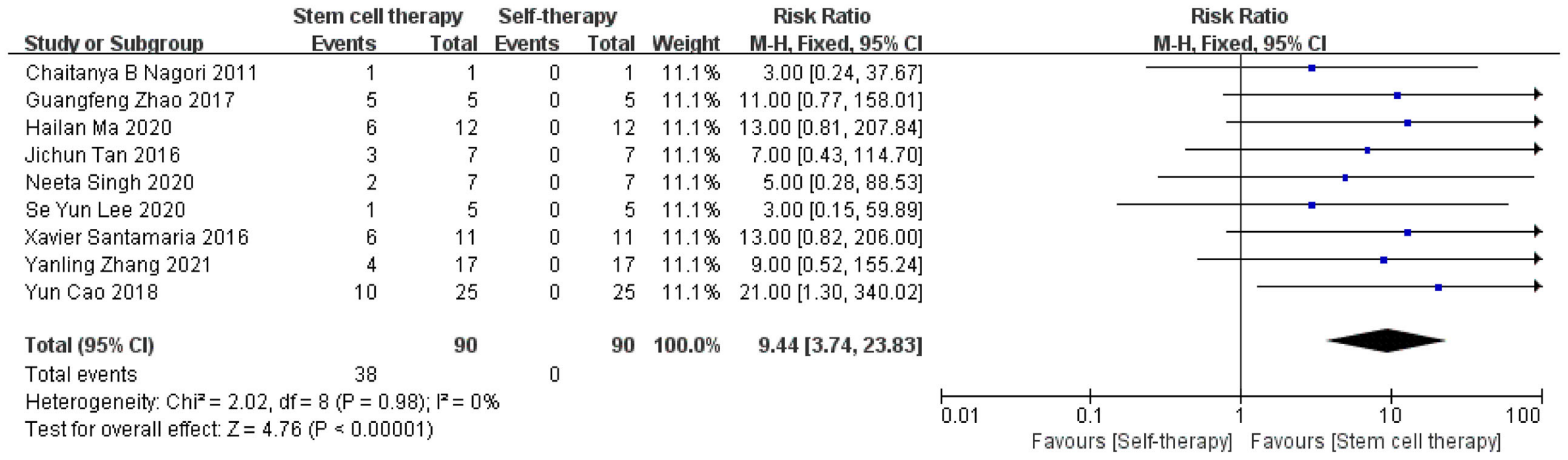
Pregnancy outcome.

Nine of the 10 studies followed a design of assessing pregnancy rates after treatment, with a total of 90 patients who had failed to conceive after traditional IUD lysis and estrogen therapy. After receiving stem cell therapy, the patients could conceive either naturally or through embryo implantation. The pregnancy rates increased significantly after stem cell therapy (**Figure 4**). There was also no obvious significant heterogeneity among studies (τ² = 0.00; χ² = 2.02, df = 8, *P =* 0.98; I² = 0%).

##### 3.4.1.3 Changes in Endometrial Thickness

**Figure 5:**
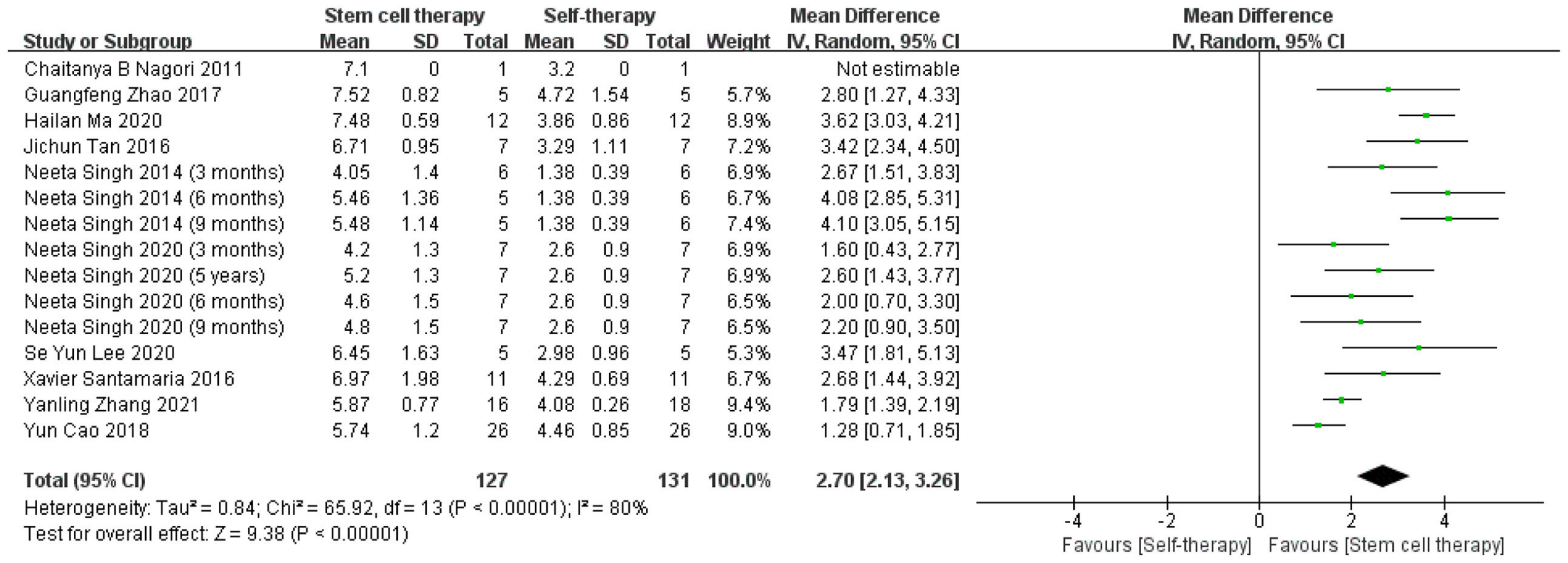
Endometrial thickness changes.

All 10 studies assessed changes in endometrial thickness, with a total of 129 patients. Although the overall efficacy of stem cell therapy on improving endometrial thickness in patients with IUA was statistically significant, heterogeneity among studies was also significant (τ² = 0.84; χ² = 65.92, df = 13, *P* < 0.00001; I² = 80%). One possible reason is the difference between autologous and allogeneic stem cell treatment, mainly owing to the uneven follow-up time (**Figure 5**).

#### 3.4.2 Comparison of the Efficacy of Autologous and Allogeneic Stem Cells

##### 3.4.2.1 Pregnancy Rates Between Autologous and Allogeneic Treatment

**Figure 6:**
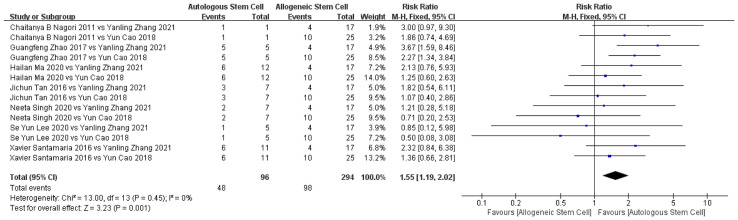
Comparison of pregnancy rate of autologous and allogeneic stem cells in treatment of IUA.

Two studies used allogeneic stem cell therapy and eight studies used autologous stem cell therapy. Among them, only seven studies mentioned pregnancy in patients at follow-up. Thus, the seven studies of autologous stem cell treatment for IUA were compared with two studies of allogeneic stem cells for IUA. The pregnancy rate with autologous stem cell therapy was significantly higher than that with allogeneic stem cell therapy. There was no significant heterogeneity among the included studies (χ²= 13, df = 13, P = 0.45; I² = 0%; **Figure 6**).

##### 3.4.2.2 Endometrial Thickness Between Autologous and Allogeneic Treatment

**Figure 7:**
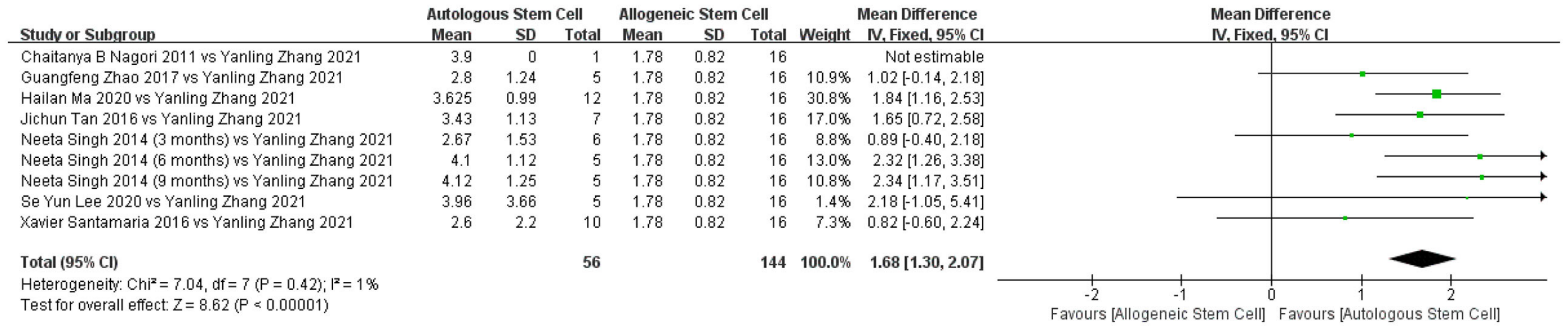
Comparison of endometrium improvement of autologous and allogeneic stem cells in treatment of IUA.

Only one allogeneic stem cell study, with 16 patients, and seven autologous stem cell studies, with 40 patients, presented detailed data on changes in endometrial thickness, enabling comparison of the efficacy of the two therapies. The endometrial thickness-repair effect of autologous stem cells in IUA patients was significantly better than that of allogeneic stem cells. In addition, there was no significant heterogeneity among the included studies (χ² = 7.04, df = 7, *P =* 0.42; I² = 1%; **Figure 7**).

### 3.5 Publication Bias and Sensitivity Analysis

**Figure 8:**
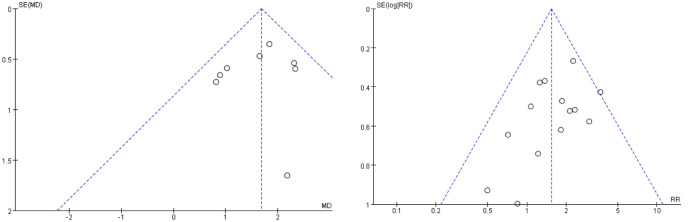
Funnel plot of endometrial thickness and pregnancy rate.

**Figure 9:**
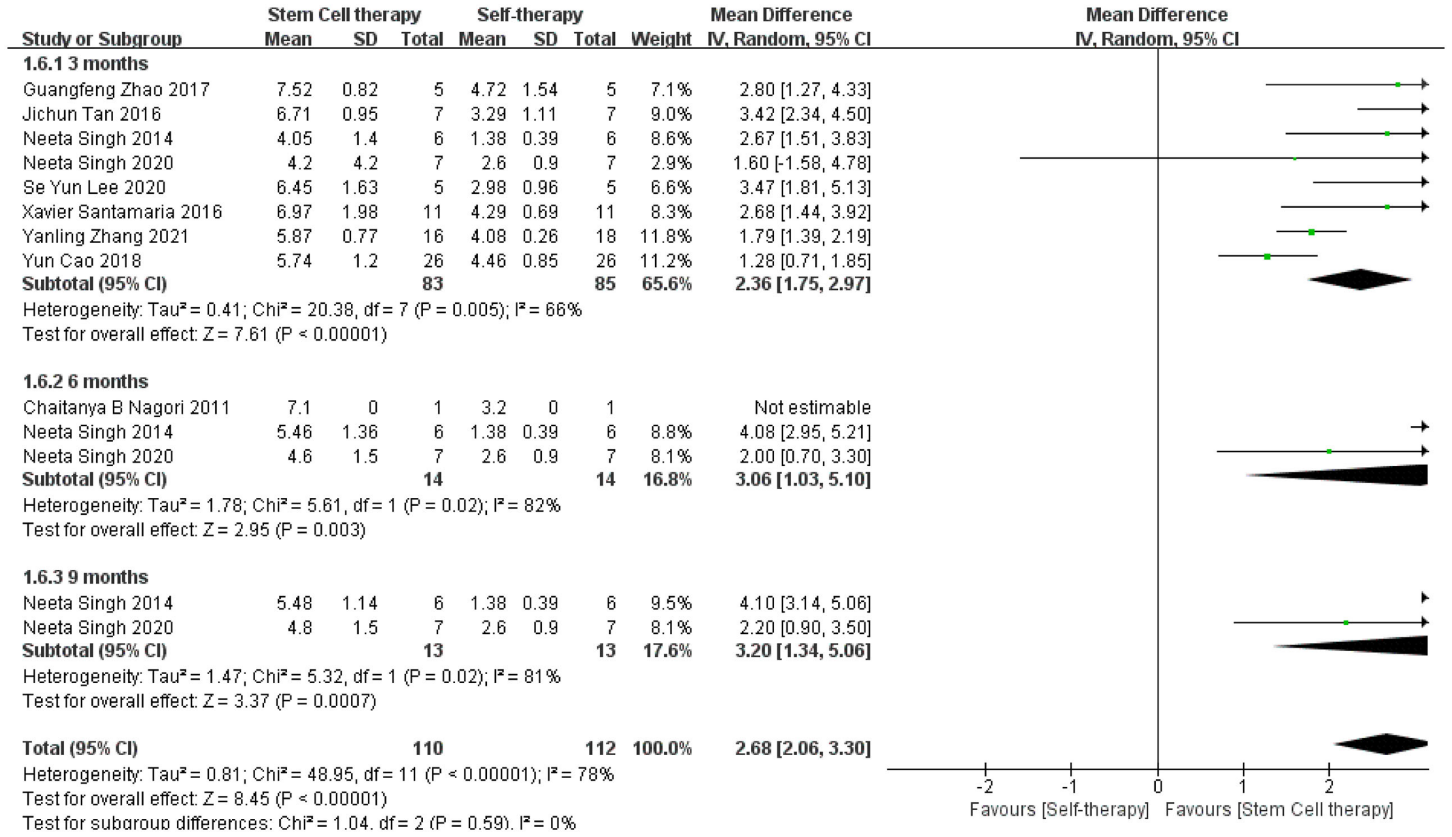
Subgroup analysis of endometrial thickness improvement.

**Figure 10:**
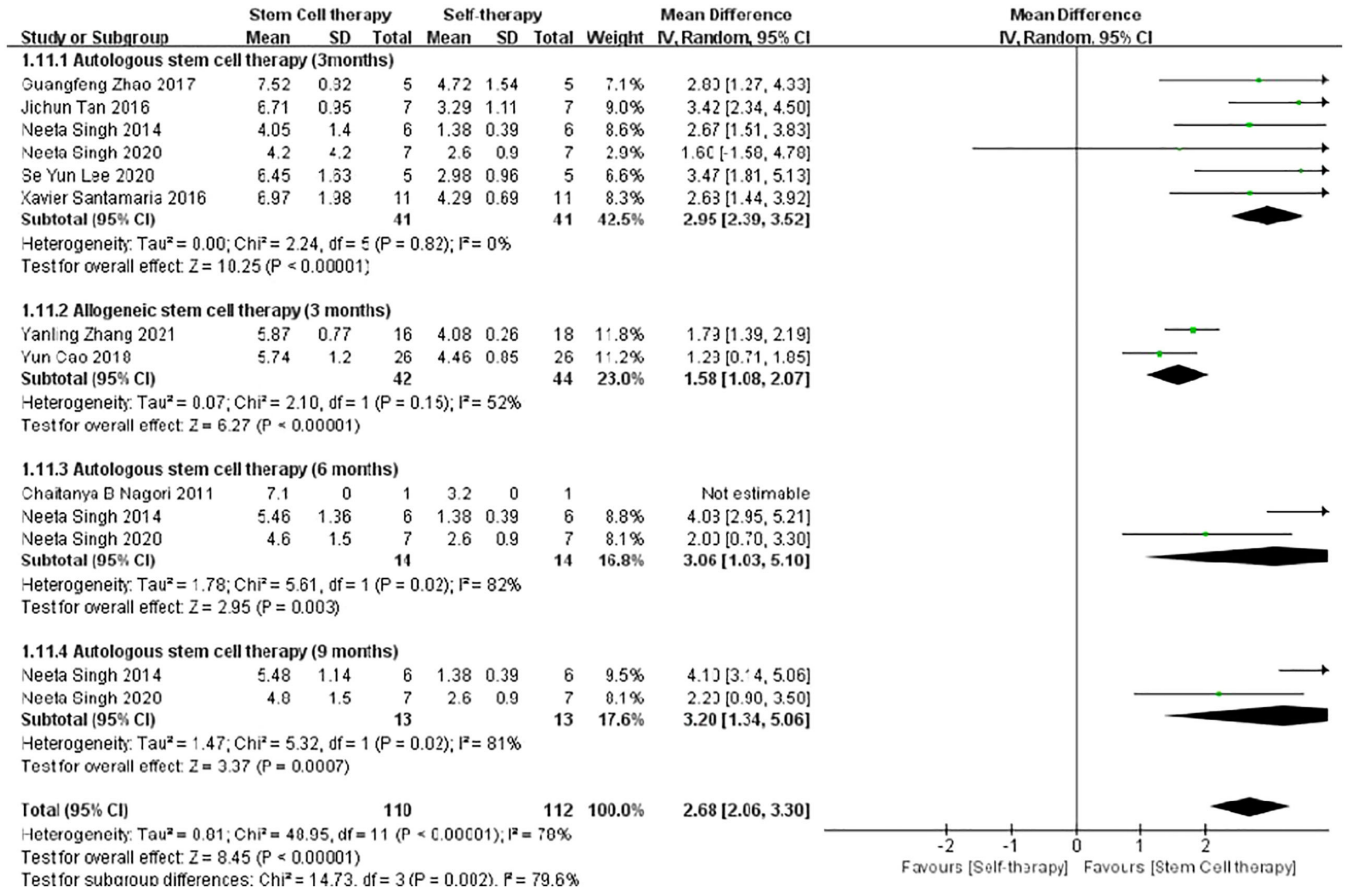
Subgroup analysis of endometrial thickness improvement with autologous stem cell and allogeneic stem cell.

The funnel plot was used to evaluate heterogeneity in improvements in endometrial thickness and pregnancy rate, along with publication bias in the comparisons between autologous and allogeneic stem cells for the treatment of IUA. The funnel plot of autologous and allogeneic stem cells was evenly distributed along the vertical direction and was symmetrical, suggesting no obvious bias or heterogeneity (**Figure 8**). Further, because stem cell therapy for IUA improved endometrial thickness with high heterogeneity in the overall efficacy evaluation, we carried out subgroup analysis according to the follow-up time. Although the improvement in endometrial thickness was statistically significant at 3, 6, and 9 months of follow-up, the heterogeneity was still high (3 months: τ² = 0.41, χ² =20.38, df = 7, *P* = 0.005, I² = 66%; 6 months: τ² = 1.78, χ² = 5.61, df = 1, *P* = 0.02, I² = 82%; 9 months: τ² = 1.47, χ² = 5.32, df = 1, *P =* 0.02), I² = 81%); thus, the specific reasons for this variability require further analysis with additional samples (**Figure 9**). Therefore, on the basis of follow-up time, we further conducted subgroup analysis according to autologous and allogeneic stem cell treatments. The results showed that the endometrial thickness of the autologous group after 3 months of follow-up was significantly thicker than that before treatment, with low heterogeneity (τ² = 0.00; χ² =2.24, df = 5, P = 0.82; I² =0%). Therefore, we considered that the high heterogeneity of endometrial thickness results in previous studies is mainly due to the combined effect of follow-up time and the use of autologous or allogeneic stem cells (**Figure 10**).

### 3.6 Safety Evaluation

Among the 10 studies (116 cases), three patients (2.59%) complained of adverse reactions during treatment, including anorexia, mild gastritis, vomiting, and abdominal colic, among others. However, these symptoms subsequently disappeared. In addition, two studies that used allogeneic stem cells to treat IUA recorded the number of white blood cells after surgery. Cao et al. (13) reported white blood cell counts of 5.96 ± 1.46 × 10^9^/L, a neutrophil percentage of 52.2 ± 8.99%, and C-reactive protein level of 2.27 ± 0.43 mg/L. Zhao et al. (14) reported a white blood cell count of 6.69 ± 1.22 × 10^9^/L, 2.37 ± 0.46 × 10^9^/L lymphocytes, and 54.76 ± 6.74% neutrophils. These results showed that the leukocyte-related indices of 44 patients after the operation were all within the normal ranges, indicating safety regardless of whether autologous stem cells or allogeneic stem cells were used for treatment. However, autologous stem cell therapy is a more ethical and acceptable treatment overall.

## 4 Discussion

In total, 10 studies with 116 patients were included in this meta-analysis. The results showed that treatment of IUA with stem cells has been generally effective and safe in improving menstruation, pregnancy rates, and endometrial thickness, although the latter results were highly heterogeneous among studies. Therefore, we divided the studies into autologous and allogeneic groups, demonstrating that autologous stem cell transplantation is significantly superior in terms of improving the pregnancy rate and endometrial thickness, with statistical significance and low heterogeneity.

In recent years, stem cells have emerged as a new treatment for IUA owing to their ability to differentiate and promote endometrial regeneration (25). Although allogeneic stem cells have the advantages of convenient extraction and large amounts at acquisition, there are still doubts about their immune-regulatory properties. Studies have shown that the application of allogeneic stem cell therapy has certain risks, including abnormal immune reconstitution, secondary tumors, and graft-versus-host disease (26). Masuda et al. (27) also pointed out that in the process of allogeneic stem cells for the treatment of immune rejection, one problem is the secondary histocompatibility antigen mismatch caused by significant immune rejection, and the other is the immune rejection mediated by natural killer cells. Although MSCs can suppress immune rejection (28), Ankrum et al. (29) pointed out that MSCs have an immune evasion capacity rather than an immune privilege property. Therefore, the use of autologous stem cells for treatment is considered to be more acceptable in clinical practice. In the two studies of allogeneic stem cell therapy (30), white blood cell counts following treatment were within the normal range. However, whether immune rejection occurs in the body after allogeneic stem cell transplantation still needs to be explored experimentally. In terms of efficacy alone, the pregnancy rate and endometrial tissue recovery in the autologous stem cell treatment group were better than those in the allogeneic stem cell treatment group.

**Figure 11:**
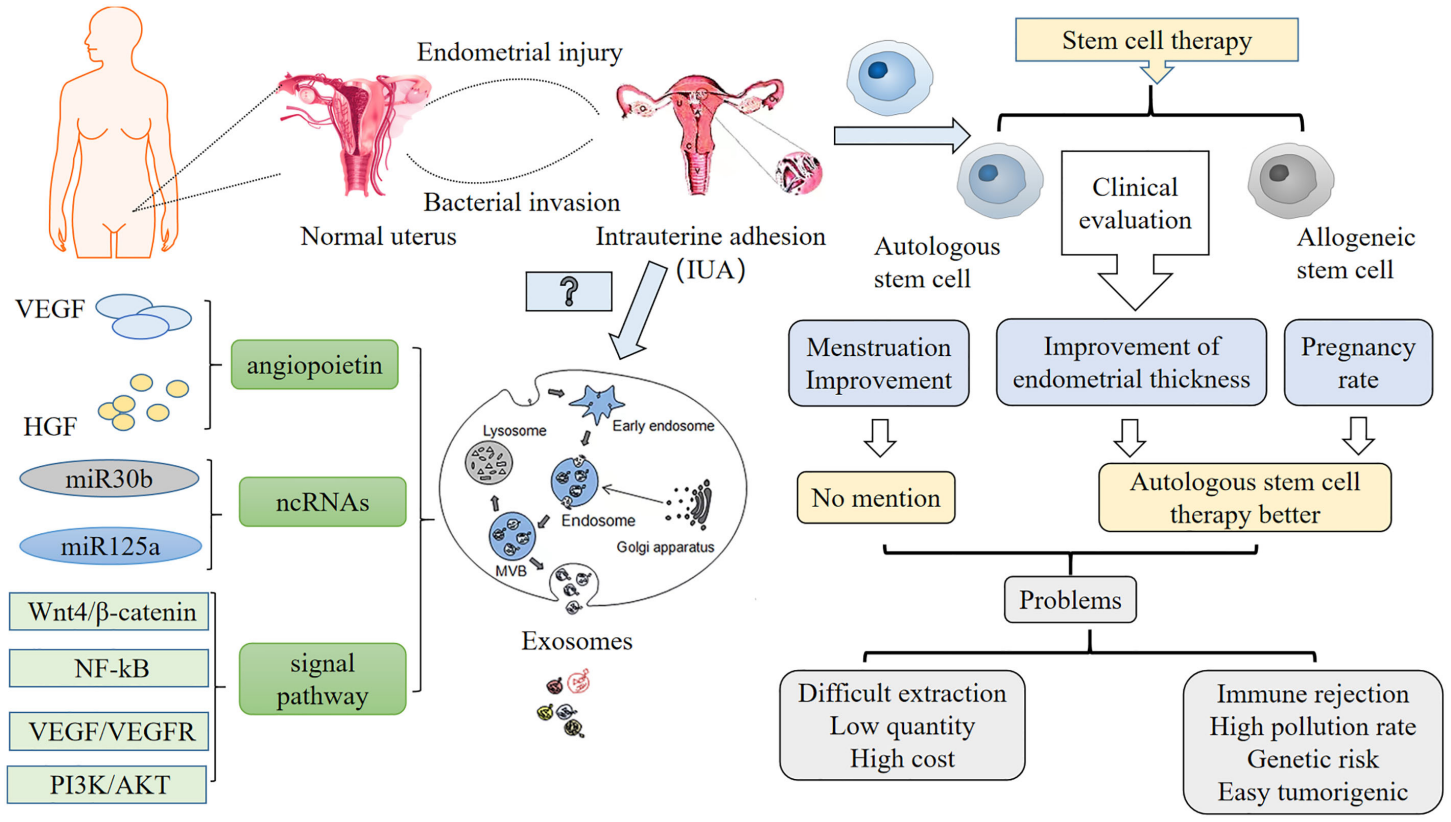
Summary of the current clinical efficacy evaluations of and existing problems with autologous and allogeneic stem cells for the treatment of intrauterine adhesions (IUAs). The results show that from the perspective of endometrial thickness improvements and pregnancy rates, autologous stem cell treatment is superior to allogeneic stem cell treatment, but both have drawbacks. Therefore, whether exosomes with lower immunogenicity can be used for the treatment of IUA in the future, to solve the problems of extraction difficulty, small numbers, and immune rejection, remains to be studied. Exosomes are extracellular vesicles released when MVBs fuse with the cell membrane or originate from cell membrane processes. Microbubbles germinate from the plasma membrane. Researchers have pointed out that exosomes mainly function through angiopoietins, such as VEGF and HGF; ncRNAs, including miR30b, miR125a; and various signaling pathways, like Wnt4/bcatenin, NF-kB, VEGF/VEGFR, and PI3K/AKT, which can promote angiogenesis. However, whether exosomes can improve IUA through these means and the mechanism through which exosomes improve IUA have still not been experimentally proven, which requires further study.

Unfortunately, the low quantity and high cost of autologous stem cell extraction still limit its clinical application. Therefore, researchers have considered whether Exos could be extracted from low-immunogenic MSCs to solve the challenges associated with using autologous and allogeneic stem cells. MSC-derived extracellular vesicles (MSC-EVs) have certain therapeutic effects on female reproductive dysfunction, such as repairing endometrial damage, inhibiting endometrial fibrosis, regulating immunity, anti-inflammatory effects, and inhibiting ovarian granulosa cell apoptosis (31), among others. Gao et al. (32) reported that the EVs secreted by BMSCs might be a promising and attractive tool to ensure the success of infertility treatment by restoring normal reproductive function. Further, integrin, leukemia inhibitory factor (LIF), and vascular endothelial growth factor (VEGF) are valuable indicators of endometrial receptivity (33). Integrin and LIF are regulators of endometrial function and play important roles in embryo implantation (34). Using a rat model, Zhao et al. (35) found that adipose tissue-derived MSC (ADSC)-Exos can maintain the normal uterine structure and promote endometrial regeneration and collagen remodeling, and could also enhance the expression of integrin-β3, LIF, and VEGF. In addition, Liao et al. (36) concluded that Exos play a role in IUA through angiopoietin, non-coding RNAs, and various signaling pathways (**Figure 11**). Therefore, Exos extracted from MSCs are expected to be a less immunogenic and more convenient method for the treatment of IUA.

In addition, estrogen, one of the classic chemotherapy drugs for the transcervical resection of adhesions (37), is helpful to prevent postoperative adhesion. In the 10 studies included in our analysis, estrogen was added as part of the treatment following hysteroscopy, and patients recovered well after the surgery. However, in a meta-analysis of three studies, estrogen therapy following hysteroscopy did not reduce IUA in any case (38). This could be related to the inconsistent evaluation standards used by different experimental groups; thus, the most appropriate design of clinical trials to unify the test result standards remains to be further explored.

This study has limitations that should be considered when interpreting the results. Incomplete effective evaluation indicators were present. Owing to the small number of research subjects with IUA scores calculated before and after treatment, the analysis was not sufficient, and thus was not included in this meta-analysis. Some of the subjects were not completely randomized and blinded, which might have improved the effectiveness of stem cell therapy for IUA, thereby affecting the results. Compared to the sample size involving autologous stem cell therapy, the allogeneic stem cell therapy group had fewer subjects, and thus the present comparison of the efficacy of the two treatments for IUA is not sufficient to conclusively indicate that autologous stem cell therapy is superior. Moreover, although the current literature indicates that Exos extracted from MSCs can improve efficacy and reduce the immune rejection caused by allogeneic stem cells, the current clinical data are insufficient to analyze the efficacy of Exos for IUA treatment. Therefore, we will continue to analyze the efficacy of Exos for the treatment of IUA using available data from animal experiments.

## 5 Conclusion

Infertility caused by the repeated recurrence of IUA remains an unsolved problem in the reproductive field. Stem cell therapy has emerged as a new treatment strategy for IUA. However, there are still immune-related issues and ethical controversies with respect to the application of autologous or allogeneic stem cell therapy. In this meta-analysis, we divided the subjects into autologous and allogeneic groups. By analyzing the overall efficacy, heterogeneity, and safety between the two groups, we found that stem cells increased the menstrual volume, prolonged menstrual time, and increased endometrial thickness and pregnancy rates after IUA treatment. However, owing to results based on the increase in endometrial thickness, we conducted a subgroup analysis and compared the autologous group with the allogeneic group. The results showed that the autologous group had a higher pregnancy rate and increased endometrial thickness. Therefore, based on the efficacy, safety, and ethics, autologous stem cell therapy is considered to be superior to allogeneic stem cells for IUA treatment. However, whether there is a difference in immune rejection between autologous and allogeneic groups remains to be further proven. We will thus continue to study the immune rejection of autologous and allogeneic stem cells in the treatment of IUA. In addition, we will further explore the role of Exos, and their effect on autologous and allogeneic stem cells in the treatment of IUA.

## Data Availability Statement

The original contributions presented in the study are included in the article/**Supplementary Material**. Further inquiries can be directed to the corresponding authors.

## Author Contributions

The envisaged role of all authors in the writing of the work is as follows: SL: 25%, Q-YS: 25%, J-MC: 25%, Q-YH: 15%, W-HC: 10%. SL and Q-YS: Funding acquisition, Project administration, Supervision, Validation, Writing-review and editing. J-MC: Roles/Writing-original draft, Writing-review and editing. Q-YH and W-HC: Writing- review and editing. All authors contributed to the article and approved the submitted version.

## Funding

This work was supported by the Science and Technology Bureau of Quanzhou (grant number 2020CT003) and Science and technology project of Fujian Provincial Health Commission (grant number 2020CXB027).

## Conflict of Interest

The authors declare that the research was conducted in the absence of any commercial or financial relationships that could be construed as a potential conflict of interest.

## Publisher’s Note

All claims expressed in this article are solely those of the authors and do not necessarily represent those of their affiliated organizations, or those of the publisher, the editors and the reviewers. Any product that may be evaluated in this article, or claim that may be made by its manufacturer, is not guaranteed or endorsed by the publisher.

## Abbreviations

ADSC, Adipose-derived mesenchymal stem cell; BMSC, Bone marrow mesenchymal stem cell; χ^2,^ Chi-square; CI, Confidence interval; Exo, Exosome; Ev, Extracellular vesicle; I^2,^ Inconsistency index; IUA, Intrauterine adhesion; LIF, Leukemia inhibitory factor; MSC, Mesenchymal stem cell; PRISMA, Preferred Reporting Items for Systematic Reviews and Meta-Analysis; RR, Relative risk; VEGF, Vascular endothelial growth factor.
