# Supplementary material for: Clinical Evaluation of Autologous and Allogeneic Stem Cell Therapy for Intrauterine Adhesions: A Systematic Review and Meta-Analysis
Source: Front Immunol. 2022 Jul 4;13:899666. doi: 10.3389/fimmu.2022.899666 (PMC9289620; doi:10.3389/fimmu.2022.899666)
Supplement: Supplementary file 1 [file DataSheet_1.docx]

Supplementary Material

| \| **DATABASE** \| **Mesh words AND Free words** \| **Results** \| \| --- \| --- \| --- \| \| PUBMED \| (((((((((((((((stem cell[MeSH Terms]) OR (Cell, Stem[Title/Abstract])) OR  (Cells, Stem[Title/Abstract])) OR (Progenitor Cells[Title/Abstract])) OR  (Cell, Progenitor[Title/Abstract])) OR (Cells, Progenitor[Title/Abstract])) OR  (Progenitor Cell[Title/Abstract])) OR (Mother Cells[Title/Abstract])) OR  (Cell, Mother[Title/Abstract])) OR (Cells, Mother[Title/Abstract])) OR  (Mother Cell[Title/Abstract])) OR (Colony-Forming Unit[Title/Abstract])) OR  (Colony Forming Unit[Title/Abstract])) OR (Colony-Forming Units[Title/Abstract])) OR  (Colony Forming Units[Title/Abstract])) AND (((((((((((((((((((((uterus synechia [MeSH Terms]) OR (Gynatresias[Title/Abstract])) OR (Asherman Syndrome[Title/Abstract])) OR (Syndrome, Asherman[Title/Abstract])) OR (Intrauterine Synechiae[Title/Abstract])) OR (Synechiae, Intrauterine[Title/Abstract])) OR (Uterine Synechiae[Title/Abstract])) OR (Synechiae, Uterine[Title/Abstract])) OR (Asherman's Syndrome[Title/Abstract])) OR (Ashermans Syndrome[Title/Abstract])) OR (Syndrome, Asherman's[Title/Abstract])) OR (intrauterine adhesion[Title/Abstract])) OR (IUA[Title/Abstract])) OR (uterine atresia[Title/Abstract])) OR (cervical atresia[Title/Abstract])) OR (uterine atrophy[Title/Abstract])) OR (sclerotic endometrium[Title/Abstract])) OR (endometrial sclerosis[Title/Abstract])) OR (intrauterine synechia[Title/Abstract])) OR (Fritsch syndrome[Title/Abstract])) OR (endometrial injury[Title/Abstract])) OR (uterine adhesion[Title/Abstract])) \| 129 \| \| Embase \| ((((((((((((((('stem cell'/exp) OR ('Cell, Stem':ab,ti) OR ('Cells, Stem':ab,ti)) OR ('Progenitor Cells':ab,ti)) OR('Cell, Progenitor':ab,ti)) OR ('Cells, Progenitor':ab,ti)) OR ('Progenitor Cell':ab,ti)) OR ('Mother Cells':ab,ti)) OR ('Cell, Mother':ab,ti)) OR ('Cells, Mother':ab,ti)) OR ('Mother Cell':ab,ti)) OR ('Colony-Forming Unit':ab,ti)) OR ('Colony Forming Unit':ab,ti)) OR ('Colony-Forming Units':ab,ti)) OR  ('Colony Forming Units':ab,ti)) AND ((((((((((((((((((((('uterus synechia'/exp) OR ('Gynatresias'/:ab,ti)) OR ('Asherman Syndrome':ab,ti)) OR ('Syndrome, Asherman':ab,ti)) OR ('Intrauterine Synechiae':ab,ti)) OR ('Synechiae, Intrauterine':ab,ti)) OR ('Uterine Synechiae':ab,ti)) OR ('Synechiae, Uterine':ab,ti)) OR ('Asherman's Syndrome':ab,ti)) OR ('Ashermans Syndrome':ab,ti)) OR ('Syndrome, Asherman's:ab,ti)) OR ('intrauterine adhesion':ab,ti)) OR ('IUA':ab,ti)) OR ('uterine atresia':ab,ti)) OR ('cervical atresia':ab,ti)) OR ('uterine atrophy':ab,ti)) OR ('sclerotic endometrium':ab,ti)) OR ('endometrial sclerosis':ab,ti)) OR ('intrauterine synechia':ab,ti)) OR ('Fritsch syndrome':ab,ti)) OR ('endometrial injury':ab,ti)) OR ('uterine adhesion':ab,ti)) \| 14 \| \| Cochrane \| (((((((((((((((MeSH descriptor:[Stem Cells] explode all trees) OR (Cell, Stem)) OR  (Cells, Stem)) OR (Progenitor Cells)) OR (Cell, Progenitor)) OR (Cells, Progenitor)) OR  (Progenitor Cell)) OR (Mother Cells)) OR (Cell, Mother)) OR (Cells, Mother)) OR (Mother Cell)) OR (Colony-Forming Unit)) OR (Colony Forming Unit)) OR (Colony-Forming Units)) OR  (Colony Forming Units)) AND (((((((((((((((((((((MeSH descriptor:[uterus synechia] explode all trees) OR (Gynatresia)) OR (Asherman Syndrome)) OR (Syndrome, Asherman)) OR (Intrauterine Synechiae)) OR (Synechiae, Intrauterine)) OR (Uterine Synechiae)) OR (Synechiae, Uterine)) OR (Asherman's Syndrome)) OR (Ashermans Syndrome)) OR (Syndrome, Asherman's)) OR (intrauterine adhesion)) OR (IUA)) OR (uterine atresia)) OR (cervical atresia)) OR (uterine atrophy)) OR (sclerotic endometrium)) OR (endometrial sclerosis)) OR (intrauterine synechia)) OR (Fritsch syndrome)) OR (endometrial injury)) OR (uterine adhesion)) \| 11 \| | |
| --- | --- | --- | --- | --- | --- | --- | --- | --- | --- | --- | --- | --- | --- |
|  |  |
